# Delirium detection in older acute medical inpatients: a multicentre prospective comparative diagnostic test accuracy study of the 4AT and the confusion assessment method

**DOI:** 10.1186/s12916-019-1367-9

**Published:** 2019-07-24

**Authors:** Susan D. Shenkin, Christopher Fox, Mary Godfrey, Najma Siddiqi, Steve Goodacre, John Young, Atul Anand, Alasdair Gray, Janet Hanley, Allan MacRaild, Jill Steven, Polly L. Black, Zoë Tieges, Julia Boyd, Jacqueline Stephen, Christopher J. Weir, Alasdair M. J. MacLullich

**Affiliations:** 1 Geriatric Medicine, Edinburgh Delirium Research Group, Royal Infirmary of Edinburgh, University of Edinburgh, Room S1642, Royal Infirmary of Edinburgh 51, Little France Crescent, Edinburgh, EH16 4SA UK; 2 Norwich Medical School, University of East Anglia, Norfolk, UK; 3 Elderly Care and Rehabilitation and Institute of Health Sciences, University of Leeds, Leeds, UK; 4 Department of Health Sciences, University of York, Hull York Medical School, Bradford District Care NHS Foundation Trust, Bradford, UK; 5 School of Health and Related Research (ScHARR), University of Sheffield, Sheffield, UK; 6 Academic Unit of Elderly Care and Rehabilitation, University of Leeds, Leeds, UK; 7 Cardiovascular Sciences and Geriatric Medicine, University of Edinburgh, Edinburgh, UK; 8 Emergency Medicine Research Group (EMERGE), NHS Lothian, Edinburgh, UK; 9 Health and Social Care, Edinburgh Napier University, Edinburgh, UK; 10 Edinburgh Clinical Trials Unit, Usher Institute of Population Health Sciences and Informatics, University of Edinburgh, Edinburgh, UK

**Keywords:** Delirium, Diagnostic test accuracy, 4AT, Confusion assessment method (CAM), Sensitivity, Specificity, Hospital

## Abstract

**Background:**

Delirium affects > 15% of hospitalised patients but is grossly underdetected, contributing to poor care. The 4 ‘A’s Test (4AT, www.the4AT.com) is a short delirium assessment tool designed for routine use without special training. The primary objective was to assess the accuracy of the 4AT for delirium detection. The secondary objective was to compare the 4AT with another commonly used delirium assessment tool, the Confusion Assessment Method (CAM).

**Methods:**

This was a prospective diagnostic test accuracy study set in emergency departments or acute medical wards involving acute medical patients aged ≥ 70. All those without acutely life-threatening illness or coma were eligible. Patients underwent (1) reference standard delirium assessment based on DSM-IV criteria and (2) were randomised to either the index test (4AT, scores 0–12; prespecified score of > 3 considered positive) or the comparator (CAM; scored positive or negative), in a random order, using computer-generated pseudo-random numbers, stratified by study site, with block allocation. Reference standard and 4AT or CAM assessments were performed by pairs of independent raters blinded to the results of the other assessment.

**Results:**

Eight hundred forty-three individuals were randomised: 21 withdrew, 3 lost contact, 32 indeterminate diagnosis, 2 missing outcome, and 785 were included in the analysis. Mean age was 81.4 (SD 6.4) years. 12.1% (95/785) had delirium by reference standard assessment, 14.3% (56/392) by 4AT, and 4.7% (18/384) by CAM. The 4AT had an area under the receiver operating characteristic curve of 0.90 (95% CI 0.84–0.96). The 4AT had a sensitivity of 76% (95% CI 61–87%) and a specificity of 94% (95% CI 92–97%). The CAM had a sensitivity of 40% (95% CI 26–57%) and a specificity of 100% (95% CI 98–100%).

**Conclusions:**

The 4AT is a short, pragmatic tool which can help improving detection rates of delirium in routine clinical care.

**Trial registration:**

International standard randomised controlled trial number (ISRCTN) 53388093. Date applied 30/05/2014; date assigned 02/06/2014.

**Electronic supplementary material:**

The online version of this article (10.1186/s12916-019-1367-9) contains supplementary material, which is available to authorized users.

## Background

Delirium is a severe neuropsychiatric syndrome, usually triggered by underlying medical illness, surgery, or drugs, which affects at least 15% of hospital inpatients [1–4]. It is more common in older people [5] and people with dementia [6]. Delirium comprises acute onset of disturbances in arousal, attention, and other domains of cognition, hallucinations, and delusions [7, 8]. Delirium is important because as well as being highly prevalent in hospitalised patients, it strongly predicts poor outcomes such as falls, other medical complications, new institutionalisation, and mortality [1, 6, 9–13]. It is also associated with patient and carer distress [14–16]. At least two thirds of cases are not identified in the emergency department and general medical settings [17–21]. The reasons for this include time constraints and lack of education and training [22–24]. Because formal psychiatric assessment for delirium diagnosis takes considerable time, guidelines and pathways advocate the use of brief assessment tools for delirium detection. Two assessment tools extensively used in clinical practice are the 4 ‘A’s Test (4AT) and the short form of the Confusion Assessment Method (CAM).

**Fig. 1:**
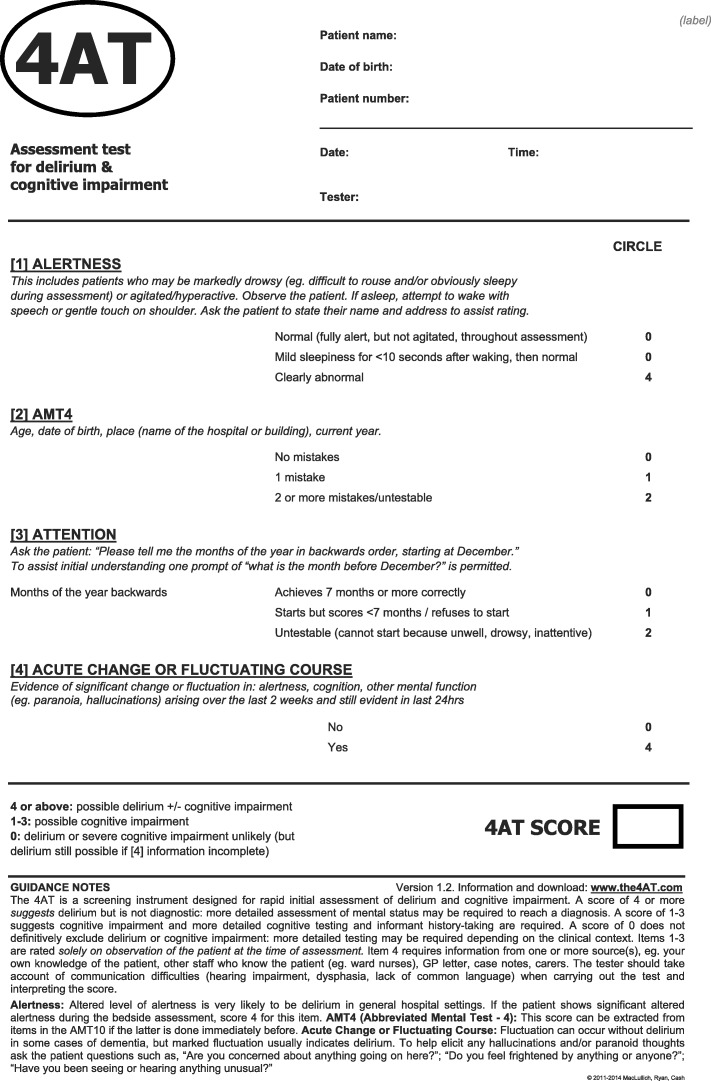
The 4 ‘A’s Test (4AT)

The 4AT [25, 26] comprises four items: (A) Alertness, (B) Abbreviated Mental Test-4, (C) Attention (Months Backwards test), and (D) acute change or fluctuating course [25, 27], Fig. 1. The 4AT was not derived directly from a single set of diagnostic criteria; rather, it has items that inform the core features of standard diagnostic criteria. It has a score range of 0–12, with scores of 4 or more (> 3) suggesting possible delirium. The structure of the 4AT is designed such that there are different ways of reaching an overall positive score (> 3). Items (A) and (D) each gives a score of 0 if negative and 4 if positive. The rationale for items (A) and (D) individually potentially triggering an overall positive 4AT score is that altered arousal and acute change are both highly specific features of delirium [28–30]. The AMT-4 (B) gives a score of 1 for one mistake and 2 for two or more mistakes or if the patient is untestable. The attention test (C) gives a score of 1 if unable to complete 7 months backwards and 2 if untestable. Therefore, patients who perform poorly or are untestable on both cognitive tests (B+C), score 4 from items (B) + (C), triggering further assessment for delirium. The rationale for the (B) and (C) scoring is that many patients with delirium are unable to undergo cognitive testing because of reduced arousal or other reasons [31, 32], and they would be unscorable or scored as negative on assessments that require cognitive testing, but the 4AT identifies that further assessment is required. The 4AT takes around 2 min and does not require special training. It is recommended in several pathways and guidelines and is in wide routine clinical use in the UK and internationally. Since publication on a dedicated website [26] in 2011, the 4AT has to date been evaluated in eight validation studies [25, 33–39] involving a total of 2577 patients, 479 with delirium. These studies have used varying designs, reference standards, clinical populations, and inclusion criteria. Sensitivities are reported as 83–100% and specificities ranging from 70 to 99%.

The CAM (short form) [28] comprises brief cognitive testing and interview followed by a four-item algorithm in which four DSM-III-R criteria for delirium are rated as being present or absent: (A) acute onset and fluctuating course, (B) inattention, (C) disorganised thinking, and (D) level of consciousness. To score positive on the CAM, both (A) and (B) must be positive, plus either or both of (C) and (D). The CAM requires specific training in rating each of the features. The cognitive testing which is carried out before completing the algorithm is not specified in the manual [40]. With the pre-algorithm interview and cognitive testing, it takes 5–10 min to complete [40]. The CAM is included in multiple international guidelines and pathways, including the UK NICE Guidelines on Delirium published in 2010 [41]. According to published systematic reviews [42–46] and a literature review carried out on 19 February 2019, the CAM has been evaluated in 22 validation studies since publication in 1990 [31, 47–66], with a total of 2437 participants (620 with delirium). As with the 4AT validation studies, these studies vary in design, population, etc. The reported range of sensitivities for delirium detection is 13–100% and the range of specificities 84–100%.

The primary objective of the present study was to conduct an evaluation of the diagnostic accuracy of the 4AT for delirium against a reference standard based on DSM-IV in patients aged 70 years and over recently admitted to hospital recruited prospectively. The secondary objective was to compare the diagnostic test accuracy of the 4AT and CAM. The rationale for performing the comparison is that the 4AT and CAM are both widely used and recommended, yet the 4AT and CAM differ in their scoring systems, and the 4AT offers potential advantages that include a shorter testing duration and no need for specific training and a process for handling untestable patients. Given these differences, it is of interest to practitioners and researchers to know if the performance of the 4AT is at least equivalent to the CAM. Additionally, both the 4AT and the CAM have been evaluated in multiple validation studies, but there are no published studies comparing the performance of these tools under the same study conditions.

## Methods

We followed the Standards for Reporting Diagnostic Accuracy (STARD) 2015 guidelines [67] for reporting diagnostic accuracy studies. The study was registered: International Standard Randomised Controlled Trial Number (ISRCTN) 53388093, UK Clinical Research Network ID: 19502, and the protocol published before database lock and statistical analysis. The objectives described in the protocol not reported here (e.g. 12-week outcomes) will be disseminated separately.

### Study design: overview

**Fig. 2:**
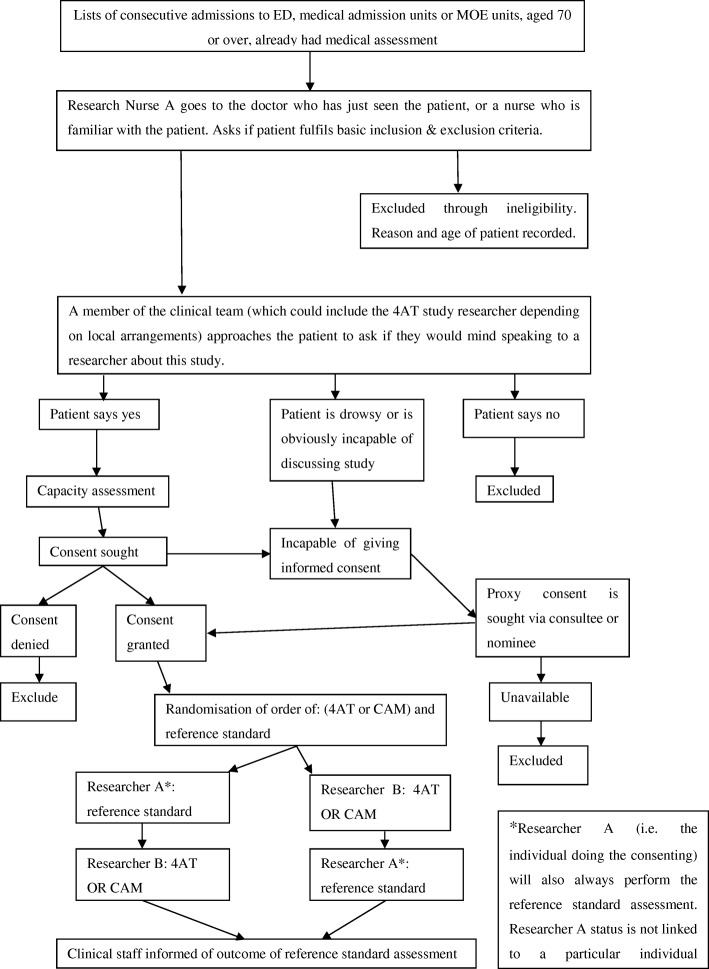
Diagnostic accuracy study: overview flowchart. ED, Emergency Department; MOE, Medicine of the Elderly; 4AT, 4 ‘A’s Test; CAM, Confusion Assessment Method

The study protocol has been published. In summary, patients aged 70 or over in emergency departments or acute general medical wards were prospectively recruited in three UK sites (Edinburgh, Bradford, and Sheffield). Each patient underwent (a) a reference standard delirium assessment lasting up to 20 min and (b) either the 4AT or the CAM. Participants were randomised to the 4AT or the CAM and also to the ordering of the reference standard and the 4AT or CAM assessment. The study flowchart is shown in Fig. 2.

### Participants

Potentially eligible participants were those without an acutely life-threatening illness or coma, in the emergency department or acute general medical wards. Initially, the recruitment windows were 4 h for the emergency department and 24 h for the acute general medical wards. Four months after the study commencement (from 19 February 2016), these were extended to 12 h and 96 h, respectively, to facilitate recruitment, particularly with respect to seeking proxy consent. The potential impact of this was explored in planned subgroup analyses.

Patients were recruited by researchers between 0800 and 2200, Monday to Friday, from eligible patients identified by the clinical team. Patients were initially approached alphabetically, then in approximately the last third of the recruitment period, through liaison with clinical staff, prioritising those at higher risk of delirium on clinical grounds (e.g. older age, likely to be admitted, higher degree of ongoing acute and chronic illnesses) to obtain a more representative sample of participants because interim analysis found a lower than expected rate of recruitment of patients who lacked capacity and thus required proxy consent. These changes to the recruitment processes were approved by the Trial Steering Committee. Informed consent was sought by trained researchers. Where the potential participant lacked the capacity to consent, recruitment proceeded under the provisions of the Mental Capacity Act, 2005, in England or Adults with Incapacity (Scotland) Act, 2000, using an appropriate personal or nominated consultee, guardian, welfare attorney, or nearest relative.

### Test methods

Researchers were nurses or trained graduate clinical research associates who underwent a systematic and detailed training process involving teaching on delirium and dementia assessment. Additionally, training on the CAM was provided according to the guidance given in the CAM instruction manual [40]. Specific training on the 4AT was not provided as the tool was designed such that this is not required. The study team had regular teleconferences to discuss the conduct of the study.

The reference standard assessment was based on DSM-IV. These diagnostic criteria were used rather than DSM-5 because the study, ethics applications, and training procedures were initiated at a similar time to publication of DSM-5, and it was not yet in use by the study team; because DSM-IV had been used in large numbers of delirium studies thus providing more direct comparability with the existing literature; and because of the concern that there was insufficient time to develop and test valid methods for reference standard assessment using DSM-5. The reference standard drew from several sources of information including all items from the Delirium Rating Scale-Revised-98 (DRS-R98) [68] and using the instructions from the manual, which include raters seeking informant history and inspection of clinical records, and a set of neuropsychological tests designed to detect core features of delirium [69, 70] comprising Observational Scale for Level of Arousal [29, 71], the Richmond Agitation-Sedation Scale [72], Digit Span [73], the Vigilance A test [74], the DelApp objective test of attention [75–77], and standard object naming and orientation questions. These assessments were used together to inform a binary diagnosis of delirium based on DSM-IV criteria. The initial diagnosis was recorded by the researcher performing the assessment for the purposes of providing immediate information to the clinical team. These initial results of the reference standard assessment were provided by this researcher to the clinical teams after the study 4AT or CAM were completed, through both an entry in the clinical notes and a verbal discussion. The final and definitive ascertainment for the study was performed later, via expert consensus from a panel comprising ZT (a psychologist), SDS (a geriatrician), and AMJM (a geriatrician), each with many years of experience of delirium assessment (> 1000 episodes individually). This final ascertainment was based on the information generated by the reference standard assessment in relation to the DSM-IV criteria, blinded to the knowledge of whether the participant had undergone the 4AT or CAM, or the results of these tests. Where there was disagreement, the panel discussed each case using the available information and reached consensus. Where the reference standard assessment results did not provide enough information to provide a definite diagnosis of delirium, the ascertainment was judged to be indeterminate.

The 4AT was scored according to the guidance notes on the 4AT form [26], using a combination of sources of available information including case notes, informants, and bedside assessment. As per the initial design of the 4AT, scores of > 3 were used to indicate possible delirium. If patients were unable to undergo cognitive testing because of reduced arousal, the cognitive items 2 and 3 were scored as ‘untestable’ and each given a score of 2 as per the 4AT guidance notes.

The CAM algorithm was scored following an interview and a set of cognitive tests, and other sources of available information including case notes and informants, as recommended in the CAM instruction manual [40]. The interview comprised general questions about the patient’s hospital stay followed by a set of cognitive tests comprising the following: days of the week backwards, counting from 20 down to 1, orientation (current day, identifying if it is day or night, current year, last meal, how long in hospital, city, name of the hospital, floor of the hospital), memory (3-word recall immediately, up to 3 trials until all 3 words recalled or 3 trials repeated; then recall at 5 min), and clock drawing. The CAM algorithm was scored as per the instruction manual. Where an item could not be assessed, for example, if the patient was unable to speak or write and thus could not undergo assessment for disorganised thinking (see instruction manual), the item was scored as negative.

The presence of dementia was sought through either a formal diagnosis of dementia in the clinical records and/or, when possible, the Informant Questionnaire on Cognitive Decline in the Elderly (IQCODE) using a cut-off score of ≥ 3.44 [78].

### Ordering of reference standard delirium assessment, 4AT and CAM

After the consent process was complete, participants were randomised in a 1:1 ratio to (a) reference standard first then either 4AT or CAM or (b) either 4AT or CAM first then reference standard via a secure online system using computer-generated pseudo-random numbers, stratified by study site, with block allocation. The reference standard assessment was performed by the researcher who conducted the capacity assessment and consenting process. A different researcher from the one performing the reference standard assessment performed either the 4AT or the CAM. Researchers performed the 4AT or the CAM according to the randomisation, with no individual researcher responsible for performing either the 4AT or the CAM; that is, each researcher performed approximately equal numbers of the 4AT and the CAM. The two assessments took place strictly within a maximum of 2 h of each other, with a target interval of 15 min. Researchers were blinded to each other’s assessments, that is, reference standard results were not available to those performing the index and comparator tests, and vice versa. The design of either 4AT or CAM rather than both 4AT and CAM being performed by each participant was chosen to avoid burden on participants, and also because the CAM testing process is longer than the 4AT and information from the CAM process could influence scoring of the 4AT, some influence of 4AT item scores on the CAM could also be possible.

### Statistical analysis

All analyses were performed using SAS version 9.3 (SAS Institute Inc., Cary, NC, USA).

#### Primary objective

We calculated positive and negative predictive values, sensitivity, and specificity for 4AT versus the reference standard. We reported the area under the receiver operating characteristic (ROC) curve and its 95% confidence interval (CI) for the 4AT.

#### Secondary objective

Comparison of 4AT and CAM: we calculated positive predictive values (PPV), negative predictive values (NPV), sensitivity, and specificity (with exact binomial 95% CI) for CAM and 4AT, and estimated the difference (4AT minus CAM) for each, assessing statistical significance of differences using Fisher’s exact test. The area under the ROC curve could not be calculated for the CAM as the outcome is binary. The overall performance of 4AT and CAM were each summarised using Youden’s Index (sensitivity minus false-positive rate) and the diagnostic odds ratio of sensitivity to specificity.

### Subgroup analyses

Predefined subgroup analyses assessed the impact of (a) time from presentation to recruitment (analysing those tested before or after 4 h (ED) or 24 h (medical admissions)) for 4AT and (b) time between index test and reference standard (analysing those tested within 30 min compared to those tested later) for both 4AT and CAM.

### Sensitivity analyses

We performed predefined sensitivity analyses where the reference standard was indeterminate by defining delirium as present and then absent. We also performed a post hoc sensitivity analysis by using the initial delirium classification recorded by the researcher at the time of the original bedside assessment (which was performed to inform clinical staff at that time). A further post hoc sensitivity analysis assumed that any patient with a missing result for the index test (4AT or CAM) had delirium.

### Missing data

If data were missing for the reference standard assessment, CAM or 4AT, or if the reference standard assessment did not yield a clear diagnosis, data from these individuals were removed from statistical analysis.

### Sample size

We planned to randomise 900 patients, 450 to assessment by 4AT and 450 to CAM. For each of 4AT and CAM, the width of the two-sided 95% confidence interval for specificity would be up to ± 0.050, and for sensitivity, up to ± 0.120. The secondary objective comparing 4AT and CAM would have 83% power to detect a difference in specificity of 0.10 and 80% power to detect a difference in sensitivity of 0.22, for a 5% two-sided significance level and analysis by continuity corrected chi-squared test.

## Results

**Fig. 3:**
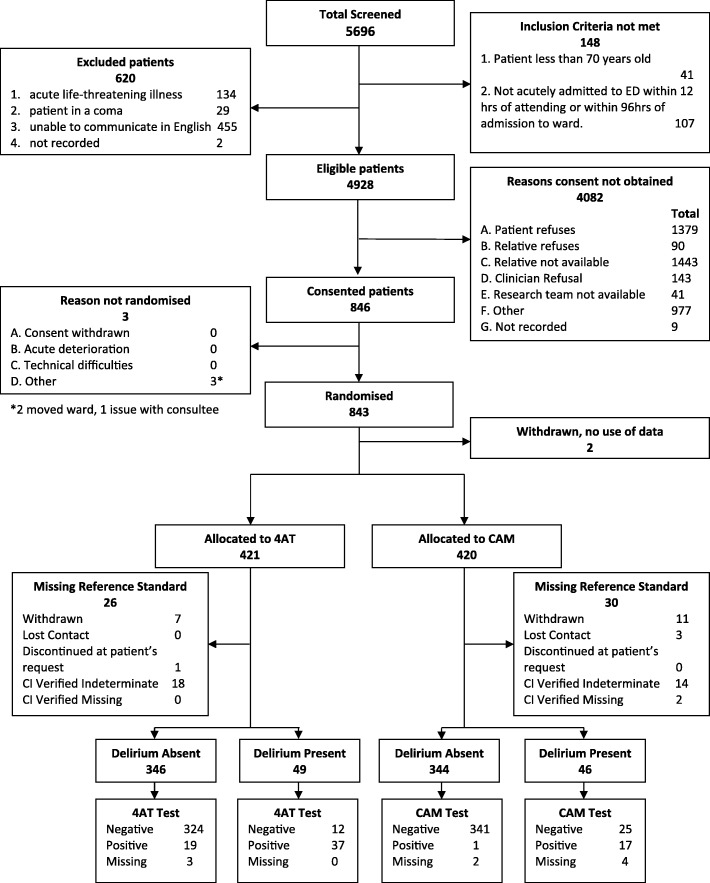
STARD diagram of the flow of participants through the study (total across all three sites)

Study recruitment commenced on 19 October 2015 and was completed on 30 December 2016, with final follow-up data collection and locking of the database on 29 June 2017. Four thousand nine hundred twenty-eight patients were eligible, from whom 843 individuals (17.1%) were recruited across the three sites and 2 withdrew before data collection, leaving 841 with data for analysis of whom 19 withdrew, 3 lost contact, 32 were classified as indeterminate from the reference standard data, and 2 had a missing outcome. Therefore, 785 individuals were included in the analyses (Fig. 3). Recruitment did not reach the target of 900 through a combination of a lower than expected rate of recruitment and a limit to the available recruitment period. However, the number recruited allowed for adequate power to test the main hypotheses as confirmed by the study statisticians and the Trial Steering Committee.

Reference standard delirium prevalence was 12.1% (*n* = 95 of 785). Individuals with delirium were older and were more likely to have dementia as documented through the clinical records or through the informant questionnaire (Table 1). Baseline characteristics for those randomised to the 4AT or CAM are shown in Additional file 1: Table S1. Reference standard delirium prevalence in those who had a valid 4AT assessment was 12.5% (*n* = 49 of 392), and in those who had a valid CAM assessment was 10.9% (*n* = 42 of 384). Delirium prevalence using the 4AT only as a diagnostic test was 14.3% (*n* = 56 of 392) and for CAM only as a diagnostic test was 4.7% (*n* = 18 of 384).

**Table 1:** Baseline demographic and clinical characteristics stratified by reference standard delirium status

	Total (*N* = 785)	Delirium present (*N* = 95)	Delirium absent (*N* = 690)	*p* value
Age (years)				
Mean (SD)	81.4 (6.4)	83.5 (6.9)	81.1 (6.3)	0.0007
Median [Q1–Q3]	81.0 [77.0–86.0]	84.0 [78.0–89.0]	81.0 [77.0–86.0]	
Gender				
Male, *n* (%)	349 (44.5%)	34 (35.8%)	315 (45.7%)	0.0697
Female, *n* (%)	436 (55.5%)	61 (64.2%)	375 (54.3%)	
Dementia diagnosis and/or IQCODE ≥ 3.44				
Yes, *n* (%)	111 (14.2%)	43 (45.3%)	68 (9.9%)	< 0.0001
No, *n* (%)	673 (85.5%)	52 (54.7%)	621 (90.1%)	
Missing*, *n* (%)	1 (0.1%)	0 (0.0%)	1 (0.1%)	
Location of first assessment				
Emergency department, *n* (%)	53 (6.8%)	10 (10.5%)	43 (6.2%)	0.2624
Acute general medical ward, *n* (%)	665 (84.7%)	76 (80.0%)	589 (85.4%)	
Hospital ward, *n* (%)	67 (8.5%)	9 (9.5%)	58 (8.4%)	

*p* value from chi-squared (categorical variables) or *t* test (continuous)

*IQCODE* Informant Questionnaire for Cognitive Impairment in the Elderly

*Missing category not included in chi-squared test

### Diagnostic test accuracy of 4AT and CAM

**Fig. 4:**
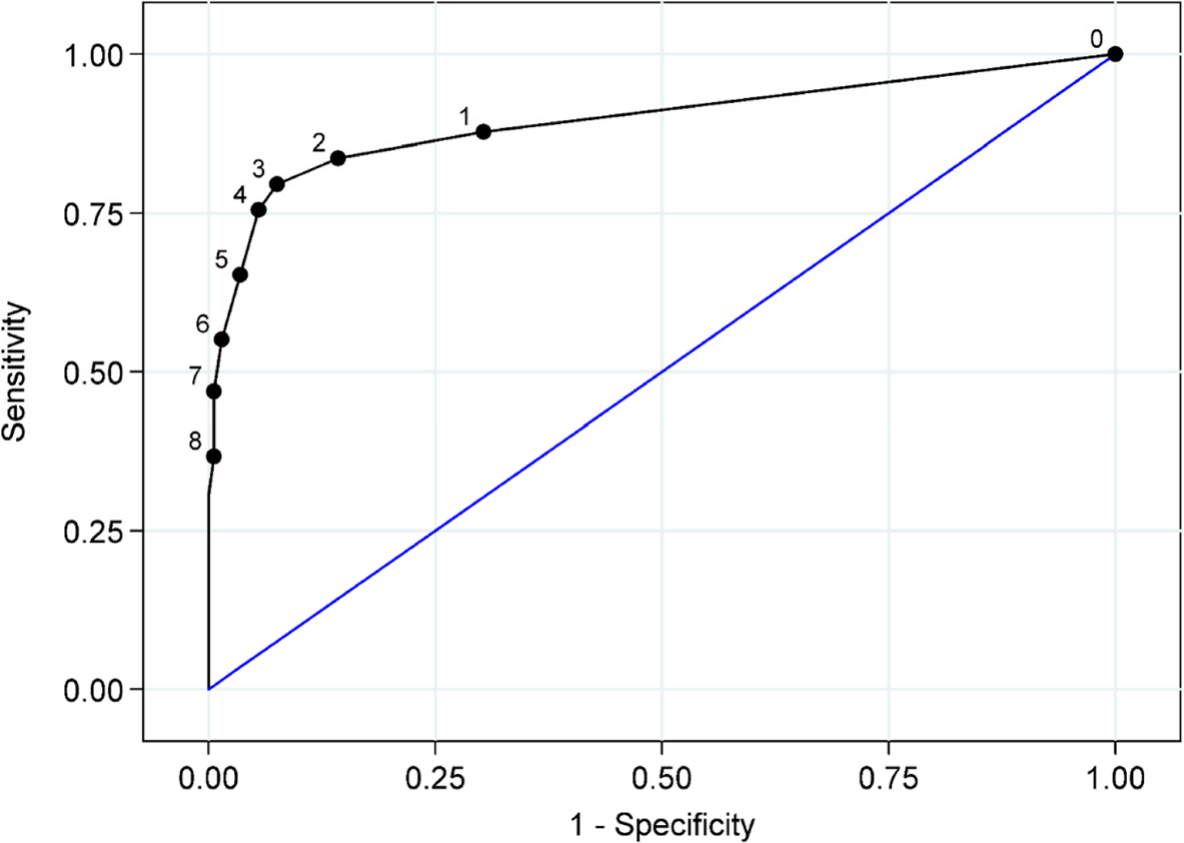
Receiver operator characteristic curve for 4AT diagnostic accuracy. 4AT scores range from 0 to 12. The cut-point of > 3 is used in the scoring scheme to denote likely delirium. The 4AT scores are considered against the reference standard delirium assessment

The main diagnostic test accuracy results for the 4AT and CAM are shown in Table 2. At a 4AT cut-off score for delirium of > 3, the sensitivity was 76% (95% CI 61 to 87%) and the specificity was 94% (95% CI 92 to 97%). The performance at different cut-off scores is shown in Additional file 2: Table S2. The area under the ROC curve for the 4AT was 0.90 (95% CI 0.84 to 0.96) (Fig. 4). The CAM had a sensitivity of 40% (95% CI 26 to 57%) and a specificity of 100% (95% CI 98 to 100%).

**Table 2:** Diagnostic test accuracy of the 4AT the CAM for diagnosis of delirium (defined by reference standard assessment)

	Sensitivity	Specificity	Positive predictive value	Negative predictive value	Youden’s Index
4AT (> 3), *n* (95% CI)	76% (61 to 87%)	94% (92 to 97%)	66% (52 to 78%)	96% (94 to 98%)	0.70
CAM Positive, *n* (95% CI)	40% (26 to 57%)	100% (98 to 100%)	94% (73 to 100%)	93% (90 to 96%)	0.40
Difference in Proportions	36% (15 to 53%)	− 6% (− 14 to 2%)	− 28% (− 53 to − 2%)	3% (− 4 to 11%)	
*p* value	0.0012	< 0.0001	0.0297	0.0629	

Numbers are estimates (95% CI). Youden’s Index is equal to sensitivity + specificity − 1, a value of zero indicates no value, and a value of 1 indicates a perfect test. The difference in proportions is 4AT-CAM for each of the tabulated measures of diagnostic accuracy, accompanied by the corresponding *p* value from Fisher’s exact test comparing proportions

*CI* confidence interval, *PPV* positive predictive value, *NPV* negative predictive value

### Subgroup analyses

There was no statistically significant difference in the diagnostic test accuracy of the 4AT between those recruited early and those recruited later after the initial presentation (Fisher’s exact test *p* values: sensitivity *p* = 0.19, specificity *p* = 0.75, PPV *p* = 0.47, NPV *p* = 0.24).

There was no statistically significant difference in the performance of either test regardless of whether or not it was performed within 30 min of the reference standard (Fisher’s exact test *p* values: sensitivity *p* = 0.16, specificity *p* = 0.24, PPV *p* = 1.00, NPV *p* = 0.56).

### Sensitivity analyses

#### Indeterminate reference standard

Assuming delirium was present for all indeterminate reference standards (*N* = 32) reduced the sensitivity of both the 4AT and CAM: 64% (95% CI 52 to 76%) and 33% (95% CI 21 to 47%), respectively (Additional file 3: Table S3). Assuming delirium was absent for all indeterminate reference standards did not substantially alter the diagnostic accuracy of the 4AT or CAM (Additional file 4: Table S4).

#### Delirium reference standard

Using the researchers’ initial reference standard assessment of delirium, the sensitivity of the 4AT was 83% (95% CI 70 to 93%) and the specificity was 94% (95% CI 91 to 96%). The sensitivity (40%; 95% CI 25 to 56%) and specificity (99%; 95% CI 98 to100%) of the CAM did not change substantially.

### Missing index test

If delirium was scored as present where the index test result was missing, this did not substantially alter the diagnostic test accuracy of the 4AT or CAM (Additional file 5: Table S5).

## Discussion

This study found that the 4AT had a sensitivity of 76% and a specificity of 94% for delirium as assessed independently by a reference standard. The area under the ROC curve was high at 0.90. These findings, in a relatively large, STARD-compliant study, provide support additional to the existing literature for the use of the 4AT as a delirium assessment instrument in clinical practice which has acceptable overall diagnostic test accuracy. The study also found that the CAM showed lower sensitivity than the 4AT, at 40%, with higher specificity, at 100%. This is the first randomised comparison of two of the most widely used delirium assessment tools in clinical practice and thus is informative for researchers with respect to their respective performance under the same study conditions.

The diagnostic test accuracy of the 4AT was broadly similar to the existing studies [25, 33–39], albeit with lower sensitivity and higher specificity than most prior studies. The difference in the sensitivity results may reflect differences in the study population, the reference standard assessment, and recruitment processes. One prior study found higher sensitivity (87%) and lower specificity (70%) and a similar area under the ROC curve of 0.84 in an unselected consecutive clinical sample using a design that did not require consenting (*N* = 434) [35]. The 4AT involves a degree of subjectivity with respect to the assessment of the level of alertness; raters are asked to rate this in a binary fashion, that is, as abnormal or normal. The reference standard assessment involves a more detailed approach to the assessment of arousal involving the three different tools: the Observational Scale of Level of Arousal, the Richmond Agitation-Sedation Scale, and the arousal element of the DelApp smartphone test of attention and arousal. It is possible that the simpler binary assessment tended to a lower level of positive score than the more complex and nuanced reference standard assessment process. Additionally, the bedside element of the 4AT (items 1–3) usually takes less than 1 min, as compared to around 20 min for the reference standard assessment. This gives more opportunity in the reference standard assessment for the observation of reduced arousal as well as fluctuation of symptoms. Further planned analyses of the present dataset will explore the relationships of individual test components of the 4AT (and the CAM) to the overall test score and components of the reference standard.

In this study, the CAM showed very high specificity and modest sensitivity for delirium. The high specificity is aligned with prior studies, the vast majority of which have found specificities of over 90%. The sensitivity of 40% was lower than in the majority of published studies. However, unlike with specificity, the literature shows notable heterogeneity in the findings with respect to CAM sensitivity, with several studies also showing lower sensitivities for the CAM [47, 49, 50, 56, 64, 66]. Differences in study populations, eligibility criteria (e.g. exclusion of drowsy patients unable to produce speech), the interview and cognitive testing performed, the training provided (this is variably described in the literature), and the background and experience of the raters may all play a role in the variability of findings [47, 58, 79]. The CAM involves binary, subjective bedside judgements of inattention, disorganised thinking, and level of consciousness; such judgements are more open to variability between raters compared to objective scoring [70, 73]. Another possible source of reduced sensitivity in some studies is that the CAM algorithm generates a negative score if disorganised thinking is not ascertained (that is, if ‘rambling, irrelevant, or incoherent speech’ [40] is not judged to be present) and if the level of consciousness is judged to be normal, though the patient may have inattention and other cognitive deficits and thus meet DSM-IV or DSM-5 criteria for delirium. Similarly, if inattention is not judged to be present but there is an altered level of consciousness, the CAM algorithm will generate a negative score.

This study had several strengths. Each participant was randomised to perform either the 4AT or CAM under the same study conditions, with the reference standard being performed independently by a different researcher. This is of interest given that the 4AT and the CAM are two of the most commonly used tools internationally. Researchers were formally trained in the use of the CAM and the reference standard assessment. The reference assessment involved gathering information from the DRS-R98, several tests of cognition, and also level of arousal. Neufeld and colleagues [80] found substantial variability in delirium reference standard assessments used in diagnostic accuracy studies of delirium assessment tools, with many not using cognitive testing as part of the assessment process. The present study had limited exclusion criteria, allowing patients with a wide spectrum of level of severity to be approached, including patients with a severely reduced level of arousal. This is pertinent because reduced level of arousal is common in emergency admissions; in one study of clinically collected data from 35,585 consecutive, unselected acute medical admissions aged > 15, 7.6% of patients had reduced level of arousal above the level of coma, and in older populations, the prevalence is higher [71, 81–83]. Given the close relationship of reduced arousal with delirium [29, 32, 71, 83], it is important that studies of delirium assessment instruments include the full spectrum of patients with reduced arousal (excluding coma). The study was relatively large and multicentre. The protocol was published in advance of database lock and analysis, and the study reporting adhered to the STARD guidelines.

Some limitations of this study should be acknowledged. In this study, only 17% of those eligible for recruitment were recruited, mostly due to patients declining to participate or no person available to provide proxy consent. The delirium rate was 12.1% according to the reference standard; prior studies have estimated that the prevalence of delirium in patients aged 70 or above at the early stages of hospital admission likely ranges from 10 to 20% [84]. The recruitment process, which required consenting (often from a proxy), may have led to a sample with a moderately lower delirium prevalence than in clinical populations. This is a known limitation of delirium studies requiring consent [85]. Most patients with delirium lack capacity, and in the context of the present study, this necessitated proxy consent and an informant to score the acute change items in the 4AT and CAM. In clinical practice, the acute change item might be informed by staff knowledge of the patient or not scored if no such information exists (though an overall positive score is still possible on the 4AT because of the scoring procedure for items (A), (B), and (C); this differs from the process that was required in the study. With respect to the reference standard, it is possible that objective assessments recorded and interpreted for this did not fully capture the researcher’s interaction with the patient and thus the researcher’s ascertainment of DSM-IV delirium features. Results from the sensitivity analysis using bedside reference standard diagnosis support this possibility, showing a higher sensitivity (83%) and a similar specificity (94%) if the researcher’s initial assessment was used. We aimed to ascertain dementia status, but it is possible that some patients had dementia but this was undiagnosed and the IQCODE was unavailable. The number of patients with known dementia was too low to allow an analysis of the performance of the 4AT or CAM in patients with and without dementia. Finally, it is possible that researcher bias may have influenced the conduct or scoring of the different index assessments (4AT or CAM) because the 4AT was designed in one of the sites of the study and involved AMJM. However, none of the researchers collecting data was involved in the development of the 4AT, the CAM was performed by researchers trained in its use as advised in the CAM instruction manual, and the reference standard was administered by researchers blind to the identity or results of the index tests.

Future studies could seek to compare the performance of the 4AT with other delirium assessment tests, such as the Single Question in Delirium (SQiD) [60], the Delirium Triage Screen [86], the brief CAM (bCAM) [86, 87], the 3D-CAM [88], and the Simple Query for Easy Evaluation of Consciousness (SQeeC) [64]. Studies could also evaluate the value of the individual items of the 4AT. This is an important issue because though ideally informant history is used to make a diagnosis of delirium, in a substantial proportion of patients, such history is not available at the point of the initial assessment or even during the inpatient stay [35, 38]. Additionally, the extent of real-world use in large clinical datasets including rates of positive scores should be evaluated. For example, the 4AT is mandated to assess for postoperative delirium in all acute hip fracture patients in the National Health Service in England, Wales, and Northern Ireland; in 2017, 86% of 63,471 patients were assessed with the 4AT, with 25% showing a positive score [89]. This is possibly an underestimate of postoperative delirium rates but suggests that the 4AT is embedded in routine clinical practice in multiple sites and likely detecting the majority of delirium across these sites. Further reporting of use of the 4AT and other tools in other large clinical datasets will be informative in determining feasibility outside of research studies.

## Conclusions

The 4AT showed moderate sensitivity, high specificity, and good overall diagnostic performance. In the present study, the 4AT showed higher sensitivity than the CAM and slightly lower specificity under the same study conditions. The CAM has been evaluated by multiple validation studies, and while many of these studies show high sensitivity, many also show that sensitivity tends to be lower where raters are not fully trained in the CAM or who lack specialist training in psychiatric assessment. Taken as a whole, the 4AT validation studies suggest that it has comparable performance to the CAM when the CAM is being performed by specially trained raters. In terms of its brevity (<2 minutes), lack of need for training, and comparable performance to the CAM, the 4AT can therefore reasonably be used as an assessment tool for delirium, particularly in clinical settings in which there is a limited time, and in which staff involved in delirium detection cannot undergo the substantial special training required for use of the CAM. Additional relevant considerations are that the 4AT can be scored if no informant history is available at the time of assessment, and also if arousal is impaired such that cognitive testing is not possible, which occurs in a substantial proportion of delirium assessments [35, 38, 64]. Given that acutely altered arousal is a highly specific indicator of delirium [29, 30, 70, 71, 83, 90–92] and that it often indicates a poor prognosis [81], a rapid provisional diagnosis of delirium with appropriate investigation and treatment  in the absence of an external informant history is reasonable. The 4AT is designed to be able to yield a positive score in patients too unwell to undergo an interview or cognitive testing [31, 32], and so no patients are classed as 'unable to assess'; this facilitates implementation and higher completion rates in clinical practice. It is important to note, however, that as with all short detection tools, a formal diagnosis of delirium in clinical practice requires assessment by a suitably qualified member of staff.

## Additional files

Additional file 1: **Table S1.** Baseline characteristics by index test (4AT or CAM). Legend: numbers are *n* (%) or mean (SD). (DOCX 13 kb)

Additional file 2: **Table S2.** Performance of various cut points of 4AT for diagnosis of delirium. Legend: numbers are estimates (95% CI). Abbreviations: CI, confidence interval; PPV, positive predictive value; NPV, negative predictive value. Youden’s Index is equal to sensitivity + specificity − 1, a value of zero indicates no value, and a value of 1 indicates a perfect test. (RTF 77 kb)

Additional file 3: **Table S3.** Sensitivity analysis of diagnostic test accuracy of 4AT versus CAM for diagnosis of delirium assuming all indeterminates are delirium present. Legend: numbers are estimate (95% CI). Difference in proportions is for 4AT-CAM. Abbreviations: CI, confidence interval; PPV, positive predictive value; NPV, negative predictive value; OR, odds ratio. Youden’s Index is equal to sensitivity + specificity − 1, a value of zero indicates no value, and a value of 1 indicates a perfect test. (DOCX 14 kb)

Additional file 4: **Table S4.** Sensitivity analysis of diagnostic test accuracy of 4AT versus CAM for diagnosis of delirium assuming all indeterminates are delirium absent. Legend: numbers are estimate (95% CI). Difference in proportions is for 4AT-CAM. Abbreviations: CI, confidence interval; PPV, positive predictive value; NPV, negative predictive value; OR, odds ratio. Youden’s Index is equal to sensitivity + specificity − 1, a value of zero indicates no value, and a value of 1 indicates a perfect test. (DOCX 14 kb)

Additional file 5: **Table S5.** Diagnostic test accuracy of 4AT versus CAM for diagnosis of delirium, assuming test scored delirium present for those with a missing 4AT or CAM score. Legend: numbers are estimate (95% CI). Difference in proportions is for 4AT-CAM. Abbreviations: CI, confidence interval; PPV, positive predictive value; NPV, negative predictive value; OR, odds ratio. Youden’s Index is equal to sensitivity + specificity − 1, a value of zero indicates no value, and a value of 1 indicates a perfect test. (DOCX 14 kb)

## Data Availability

Analyses of the data in this study are still ongoing. We shall make fully anonymised data available on the website https://datashare.is.ed.ac.uk/ in an estimated 1 year from the publication of this manuscript.

## Abbreviations

3D-CAM: 3-Minute diagnostic assessment for delirium using the Confusion Assessment Method
4AT: 4 ‘A’s Test
bCAM: Brief Confusion Assessment Method
CAM: Confusion Assessment Method
CI: Confidence interval
DSM-5: Diagnostic and Statistical Manual, 5th edition
DSM-III-R: Diagnostic and Statistical Manual, 3rd edition, revised
DSM-IV: Diagnostic and Statistical Manual, 4th edition
ED: Emergency department
IQCODE: Informant Questionnaire on Cognitive Decline in the Elderly
ISRCTN: International Standard Randomised Controlled Trial Number
NHS: National Health Service
NIHR HTA: National Institute of Health Research Health Technology Assessment Programme
NPV: Negative predictive value
PPV: Positive predictive value
REC: Research ethics committee
ROC: Receiver operating characteristic
SqeeC: Simple Query for Easy Evaluation of Consciousness
SQiD: Single Question in Delirium
STARD: Standards for Reporting Diagnostic Accuracy

## References

[1] Reynish EL, Hapca SM, De Souza N, Cvoro V, Donnan PT, Guthrie B (2017). Epidemiology and outcomes of people with dementia, delirium, and unspecified cognitive impairment in the general hospital: prospective cohort study of 10,014 admissions. BMC Med.

[2] Pendlebury ST, Lovett NG, Smith SC, Dutta N, Bendon C, Lloyd-Lavery A, Mehta Z, Rothwell PM (2015). Observational, longitudinal study of delirium in consecutive unselected acute medical admissions: age-specific rates and associated factors, mortality and re-admission. BMJ open.

[3] Marcantonio ER (2017). Delirium in hospitalised older adults. N Engl J Med.

[4] Oh ES, Fong TG, Hshieh TT, Inouye SK (2017). Delirium in older persons: advances in diagnosis and treatment. JAMA.

[5] Goldberg SE, Whittamore KH, Harwood RH, Bradshaw LE, Gladman JR, Jones RG (2012). The prevalence of mental health problems among older adults admitted as an emergency to a general hospital. Age Ageing.

[6] The Royal College of Psychiatrists (2005). Who cares wins.

[7] American Psychiatric Association. Diagnostic and statistical manual of mental disorders: DSM-IV-TR. Washington, DC: American Psychiatric Association; 2000.

[8] American Psychiatric Association. Diagnostic and statistical manual of mental disorders: 5th ed. Washington, DC: American Psychiatric Association; 2013.

[9] Davis DH, Muniz-Terrera G, Keage HA, Stephan BC, Fleming J, Ince PG, Matthews FE, Cunningham C, Ely EW, MacLullich AM (2017). Association of delirium with cognitive decline in late life: a neuropathologic study of 3 population-based cohort studies. JAMA Psychiatry.

[10] MacLullich AMJ, Beaglehole A, Hall RJ, Meagher DJ (2009). Delirium and long-term cognitive impairment. Int Rev Psychiatry.

[11] Witlox J, Eurelings LSM, de Jonghe JFM, Kalisvaart KJ, Eikelenboom P, van Gool WA (2010). Delirium in elderly patients and the risk of postdischarge mortality, institutionalisation, and dementia: a meta-analysis. JAMA.

[12] Han JH, Shintani A, Eden S, Morandi A, Solberg LM, Schnelle J, Dittus RS, Storrow AB, Ely EW (2010). Delirium in the emergency department: an independent predictor of death within 6 months. Ann Emerg Med.

[13] Burton JK, Guthrie B, Hapca SM, Cvoro V, Donnan PT, Reynish EL (2018). Living at home after emergency hospital admission: prospective cohort study in older adults with and without cognitive spectrum disorder. BMC Med.

[14] Partridge JS, Martin FC, Harari D, Dhesi JK (2013). The delirium experience: what is the effect on patients, relatives and staff and what can be done to modify this?. Int J Geriatr Psychiatry.

[15] Martins S, Pinho E, Correia R, Moreira E, Lopes L, Paiva JA, Azevedo L, Fernandes L (2018). What effect does delirium have on family and nurses of older adult patients?. Ageing Ment Health.

[16] Racine AM, D’Aquila M, Schmitt EM, Gallagher J, Marcantonio ER, Jones RN, Inouye SK, Schulman-Green D, Group BS (2019). Delirium burden in patients and family caregivers: development and testing of new instruments. Gerontologist.

[17] Collins N, Blanchard MR, Tookman A, Sampson EL (2010). Detection of delirium in the acute hospital. Age Ageing.

[18] Han JH, Zimmerman EE, Cutler N, Schnelle J, Morandi A, Dittus RS, Storrow AB, Ely EW (2009). Delirium in older emergency department patients: recognition, risk factors, and psychomotor subtypes. Acad Emerg Med.

[19] Traynor V, Cordato N, Burns P, Xu Y, Britten N, Duncan K, DeVries L, McKinnon C (2016). Is delirium being detected in emergency?. Australas J Ageing.

[20] Bellelli G, Nobili A, Annoni G, Morandi A, Djade CD, Meagher DJ, Maclullich AM, Davis D, Mazzone A, Tettamanti M (2015). Under-detection of delirium and impact of neurocognitive deficits on in-hospital mortality among acute geriatric and medical wards. Eur J Intern Med.

[21] Stelmokas J, Gabel N, Flaherty JM, Rayson K, Tran K, Anderson JR, Bieliauskas LA (2016). Delirium detection and impact of comorbid health conditions in a post-acute rehabilitation hospital setting. PLoS One.

[22] Davis D, MacLullich A (2009). Understanding barriers to delirium care: a multicentre survey of knowledge and attitudes among UK junior doctors. Age Ageing.

[23] Fisher JM, Gordon AL, MacLullich AM, Tullo E, Davis DH, Blundell A, Field RH, Teodorczuk A (2015). Towards an understanding of why undergraduate teaching about delirium does not guarantee gold-standard practice--results from a UK national survey. Age Ageing.

[24] Teodorczuk A, Reynish E, Milisen K (2012). Improving recognition of delirium in clinical practice: a call for action. BMC Geriatr.

[25] Bellelli G, Morandi A, Davis DH, Mazzola P, Turco R, Gentile S, Ryan T, Cash H, Guerini F, Torpilliesi T (2014). Validation of the 4AT, a new instrument for rapid delirium screening: a study in 234 hospitalised older people. Age Ageing.

[26] The 4 “A”s Test . www.the4AT.com. Accessed 1 Sept 2014.

[27] Shenkin SD, Fox C, Godfrey M, Siddiqi N, Goodacre S, Young J, Anand A, Grey A, Smith J, Ryan T (2018). Protocol for validation of the 4AT, a rapid screening tool for delirium: a multicentre prospective diagnostic test accuracy study. BMJ Open.

[28] Inouye SK, van Dyck CH, Alessi CA, Balkin S, Siegal AP, Horwitz RI (1990). Clarifying confusion: the confusion assessment method. A new method for detection of delirium. Ann Intern Med.

[29] Tieges Z, McGrath A, Hall RJ, Maclullich AM (2013). Abnormal level of arousal as a predictor of delirium and inattention: an exploratory study. Am J Geriatr Psychiatry.

[30] Chester JG, Harrington MB, Rudolph JL, Grp VADW (2012). Serial administration of a modified Richmond agitation and sedation scale for delirium screening. J Hosp Med.

[31] Yates C, Stanley N, Cerejeira JM, Jay R, Mukaetova-Ladinska EB (2009). Screening instruments for delirium in older people with an acute medical illness. Age Ageing.

[32] European Delirum Association, American Delirium Society (2014). The DSM-5 criteria, level of arousal and delirium diagnosis: inclusiveness is safer. BMC Med.

[33] Lees R, Corbet S, Johnston C, Moffitt E, Shaw G, Quinn TJ (2013). Test accuracy of short screening tests for diagnosis of delirium or cognitive impairment in an acute stroke unit setting. Stroke.

[34] Kuladee S, Prachason T (2016). Development and validation of the Thai version of the 4 ‘A’s Test for delirium screening in hospitalised elderly patients with acute medical illnesses. Neuropsychiatr Dis Treat.

[35] Hendry K, Quinn TJ, Evans J, Scortichini V, Miller H, Burns J, Cunnington A, Stott DJ (2016). Evaluation of delirium screening tools in geriatric medical inpatients: a diagnostic test accuracy study. Age Ageing.

[36] De J, Wand AP, Smerdely PI, Hunt GE (2017). Validating the 4A’s test in screening for delirium in a culturally diverse geriatric inpatient population. Int J Geriatr Psychiatry.

[37] Infante MT, Pardini M, Balestrino M, Finocchi C, Malfatto L, Bellelli G, Mancardi GL, Gandolfo C, Serrati C (2017). Delirium in the acute phase after stroke: comparison between methods of detection. Neurol Sci.

[38] O’Sullivan D, Brady N, Manning E, O’Shea E, O’Grady S, O’Regan N, Timmons S (2018). Validation of the 6-Item Cognitive Impairment Test and the 4AT test for combined delirium and dementia screening in older emergency department attendees. Age Ageing.

[39] Saller T, MacLullich A, Schaher T, Crispin A, Neitzert R, Schule C, Von Dossow V, Hofmann-Kiefer KF: Validation study of the 4 ‘A’s Test (4AT) for delirium detection in post-anaesthesia care. Anaesthesia 2019,[Epub]. 10.1111/anae.14682 31038212

[40] Inouye SK (2014). The Short Confusion Assessment Method (Short CAM): training manual and coding guide.

[41] Delirium: prevention, diagnosis and management. https://www.nice.org.uk/guidance/cg103. Accessed 1 Sept 2014.

[42] Wei LA, Fearing MA, Sternberg EJ, Inouye SK (2008). The Confusion Assessment Method: a systematic review of current usage. J Am Geriatr Soc.

[43] Wong CL, Holroyd-Leduc J, Simel DL, Straus SE (2010). Does this patient have delirium?: value of bedside instruments. JAMA.

[44] Shi Q, Warren L, Saposnik G, Macdermid JC (2013). Confusion assessment method: a systematic review and meta-analysis of diagnostic accuracy. Neuropsychiatr Dis Treat.

[45] De J, Wand AP (2015). Delirium screening: a systematic review of delirium screening tools in hospitalised patients. Gerontologist.

[46] van Velthuijsen EL, Zwakhalen SM, Warnier RM, Mulder WJ, Verhey FR, Kempen GI (2016). Psychometric properties and feasibility of instruments for the detection of delirium in older hospitalised patients: a systematic review. Int J Geriatr Psychiatry.

[47] Rockwood K, Cosway S, Stolee P, Kydd D, Carver D, Jarrett P, O’Brien B (1994). Increasing the recognition of delirium in elderly patients. J Am Geriatr Soc.

[48] Farrell KR, Ganzini L (1995). Misdiagnosing delirium as depression in medically ill elderly patients. Arch Intern Med.

[49] Pompei P, Foreman M, Cassel CK, Alessi C, Cox D (1995). Detecting delirium among hospitalised older patients. Arch Intern Med.

[50] Rolfson DB, McElhaney JE, Jhangri GS, Rockwood K (1999). Validity of the confusion assessment method in detecting postoperative delirium in the elderly. Int Psychogeriatr.

[51] Fabbri RM, Moreira MA, Garrido R, Almeida OP (2001). Validity and reliability of the Portuguese version of the Confusion Assessment Method (CAM) for the detection of delirium in the elderly. Arq Neuropsiquiatr.

[52] Laurila JV, Pitkala KH, Strandberg TE, Tilvis RS (2002). Confusion assessment method in the diagnostics of delirium among aged hospital patients: would it serve better in screening than as a diagnostic instrument?. Int J Geriatr Psychiatry.

[53] Gonzalez M, de Pablo J, Fuente E, Valdes M, Peri JM, Nomdedeu M, Matrai S (2004). Instrument for detection of delirium in general hospitals: adaptation of the confusion assessment method. Psychosomatics.

[54] Gaudreau JD, Gagnon P, Harel F, Tremblay A, Roy MA (2005). Fast, systematic, and continuous delirium assessment in hospitalised patients: the nursing delirium screening scale. J Pain Symptom Manage.

[55] Leung J, Leung V, Leung CM, Pan PC (2008). Clinical utility and validation of two instruments (the Confusion Assessment Method algorithm and the Chinese version of Nursing Delirium Screening Scale) to detect delirium in geriatric inpatients. Gen Hosp Psychiatry.

[56] Radtke FM, Franck M, Schneider M, Luetz A, Seeling M, Heinz A, Wernecke KD, Spies CD (2008). Comparison of three scores to screen for delirium in the recovery room. Br J Anaesth.

[57] Hestermann U, Backenstrass M, Gekle I, Hack M, Mundt C, Oster P, Thomas C (2009). Validation of a German version of the Confusion Assessment Method for delirium detection in a sample of acute geriatric patients with a high prevalence of dementia. Psychopathology.

[58] Ryan K, Leonard M, Guerin S, Donnelly S, Conroy M, Meagher D (2009). Validation of the confusion assessment method in the palliative care setting. Palliat Med.

[59] Radtke FM, Franck M, Schust S, Boehme L, Pascher A, Bail HJ, Seeling M, Luetz A, Wernecke KD, Heinz A (2010). A comparison of three scores to screen for delirium on the surgical ward. World J Surg.

[60] Sands MB, Dantoc BP, Hartshorn A, Ryan CJ, Lujic S (2010). Single Question in Delirium (SQiD): testing its efficacy against psychiatrist interview, the Confusion Assessment Method and the Memorial Delirium Assessment Scale. Palliat Med.

[61] Wongpakaran N, Wongpakaran T, Bookamana P, Pinyopornpanish M, Maneeton B, Lerttrakarnnon P, Uttawichai K, Jiraniramai S (2011). Diagnosing delirium in elderly Thai patients: utilisation of the CAM algorithm. BMC Fam Pract.

[62] Thomas C, Kreisel SH, Oster P, Driessen M, Arolt V, Inouye SK (2012). Diagnosing delirium in older hospitalised adults with dementia: adapting the confusion assessment method to international classification of diseases, tenth revision, diagnostic criteria. J Am Geriatr Soc.

[63] Charoensak S, Thunmanurukkit A, Sittironnarit G, Sartra T (2014). Validity and reliability of the Thai version of the confusion assessment method. J Med Assoc Thai.

[64] Lin HS, Eeles E, Pandy S, Pinsker D, Brasch C, Yerkovich S (2015). Screening in delirium: a pilot study of two screening tools, the Simple Query for Easy Evaluation of Consciousness and Simple Question in Delirium. Australas J Ageing.

[65] Martins S, Lourenco C, Pinto-de-Sousa J, Conceicao F, Paiva JA, Simoes MR, Fernandes L (2015). Validation study of the European Portuguese version of the Confusion Assessment Method (CAM). Int Psychogeriatr.

[66] Smulter N, Lingehall HC, Gustafson Y, Olofsson B, Engstrom KG (2015). Validation of the confusion assessment method in detecting postoperative delirium in cardiac surgery patients. Am J Crit Care.

[67] Cohen JF, Korevaar DA, Altman DG, Bruns DE, Gatsonis CA, Hooft L, Irwig L, Levine D, Reitsma JB, de Vet HC (2016). STARD 2015 guidelines for reporting diagnostic accuracy studies: explanation and elaboration. BMJ Open.

[68] Trzepacz PT, Mittal D, Torres R, Kanary K, Norton J, Jimerson N (2001). Validation of the Delirium Rating Scale-revised-98: comparison with the Delirium Rating Scale and the Cognitive Test for Delirium. J Neuropsychiatry Clin Neurosci.

[69] Tieges Z, Brown LJ, MacLullich AM (2014). Objective assessment of attention in delirium: a narrative review. Int J Geriatr Psychiatry.

[70] Tieges Z, Evans JJ, Neufeld KJ, MacLullich AM (2018). The neuropsychology of delirium: advancing the science of delirium assessment. Int J Geriatr Psychiatry.

[71] Richardson SJ, Davis DHJ, Bellelli G, Hasemann W, Meagher D, Kreisel SH, MacLullich AMJ, Cerejeira J, Morandi A (2017). Detecting delirium superimposed on dementia: diagnostic accuracy of a simple combined arousal and attention testing procedure. Int Psychogeriatr.

[72] Sessler CN, Gosnell MS, Grap MJ, Brophy GM, O’Neal PV, Keane KA, Tesoro EP, Elswick RK (2002). The Richmond Agitation-Sedation Scale: validity and reliability in adult intensive care unit patients. Am J Respir Crit Care Med.

[73] Simon SE, Bergmann MA, Jones RN, Murphy KM, Orav EJ, Marcantonio ER (2006). Reliability of a structured assessment for nonclinicians to detect delirium among new admissions to postacute care. J Am Med Dir Assoc.

[74] Hart RP, Levenson JL, Sessler CN, Best AM, Schwartz SM, Rutherford LE (1996). Validation of a cognitive test for delirium in medical ICU patients. Psychosomatics.

[75] Tieges Z, Stiobhairt A, Scott K, Suchorab K, Weir A, Parks S, Shenkin S, MacLullich A (2015). Development of a smartphone application for the objective detection of attentional deficits in delirium. Int Psychogeriatr.

[76] Tang E, Laverty M, Weir A, Wilson ES, Walsh TS, Allerhand M, Parks S, MacLullich AMJ, Tieges Z (2018). Development and feasibility of a smartphone-based test for the objective detection and monitoring of attention impairments in delirium in the ICU. J Crit Care.

[77] Rutter LM, Nouzova E, Stott DJ, Weir CJ, Assi V, Barnett JH, Clarke C, Duncan N, Evans J, Green S (2018). Diagnostic test accuracy of a novel smartphone application for the assessment of attention deficits in delirium in older hospitalised patients: a prospective cohort study protocol. BMC Geriatr.

[78] Neufeld KJ, Nelliot A, Inouye SK, Ely EW, Bienvenu OJ, Lee HB, Needham DM (2014). Delirium diagnosis methodology used in research: a survey-based study. Am J Geriatr Psychiatry.

[79] Inouye SK, Foreman MD, Mion LC, Katz KH, Cooney LM (2001). Nurses’ recognition of delirium and its symptoms - comparison of nurse and researcher ratings. Arch Intern Med.

[80] Wozniak AW, Colantuoni EJ, Schreiber MP, Neufeld KJ, Needham DM (2015). Corticosteroids and transition to delirium in acute lung injury: multinomial logistic regression analysis accounting for multiple States. Crit Care Med.

[81] Todd A, Blackley S, Burton JK, Stott DJ, Ely EW, Tieges Z, MacLullich AMJ, Shenkin SD (2017). Reduced level of arousal and increased mortality in adult acute medical admissions: a systematic review and meta-analysis. BMC Geriatr.

[82] Bellelli G, Mazzone A, Morandi A, Latronico N, Perego S, Zazzetta S, Mazzola P, Annoni G (2016). The effect of an impaired arousal on short- and long-term mortality of elderly patients admitted to an acute geriatric unit. J Am Med Dir Assoc.

[83] Morandi A, Han JH, Meagher D, Vasilevskis E, Cerejeira J, Hasemann W, MacLullich AM, Annoni G, Trabucchi M, Bellelli G (2016). Detecting delirium superimposed on dementia: evaluation of the diagnostic performance of the Richmond Agitation and Sedation Scale. J Am Med Dir Assoc.

[84] Han JH, Wilson A, Ely EW (2010). Delirium in the older emergency department patient: a quiet epidemic. Emerg Med Clin North Am.

[85] Adamis D, Martin FC, Treloar A, Macdonald AJD (2005). Capacity, consent, and selection bias in a study of delirium. J Med Ethics.

[86] Han JH, Wilson A, Vasilevskis EE, Shintani A, Schnelle JF, Dittus RS, Graves AJ, Storrow AB, Shuster J, Ely EW (2013). Diagnosing delirium in older emergency department patients: validity and reliability of the delirium triage screen and the brief confusion assessment method. Ann Emerg Med.

[87] Han JH, Wilson A, Vasilevskis EE, Storrow AB, Shintani A, Schnelle J, Graves AJ, Dittus RS, Ely EW (2012). The validation of the emergency department Delirium Triage Screen in older emergency department patients. Ann Emerg Med.

[88] Marcantonio ER, Ngo LH, O’Connor M, Jones RN, Crane PK, Metzger ED, Inouye SK (2014). 3D-CAM: derivation and validation of a 3-min diagnostic interview for CAM-defined delirium: a cross-sectional diagnostic test study. Ann Int Med.

[89] National Hip Fracture Database (NHFD), Annual Report 2018. https://www.nhfd.co.uk/files/2018ReportFiles/NHFD-2018-Annual-Report-v101.pdf. Accessed 1 Mar 2019.

[90] Lagarto L, Albuquerque E, Loureiro D, Vieira F, Esteves P, Neves S, Teixeira-Verissimo M, Cerejeira J. Arousal changes and delirium in acute medically-ill male older patients with and without dementia: a prospective study during hospitalisation. Ageing Ment Health. 2018:1–8. [Epub ahead of print] 10.1080/13607863.2018.1548569 30595038

[91] Han JH, Vasilevskis EE, Schnelle JF, Shintani A, Dittus RS, Wilson A, Ely EW (2015). The diagnostic performance of the Richmond Agitation Sedation Scale for detecting delirium in older emergency department patients. Acad Emerg Med.

[92] Grossmann FF, Hasemann W, Kressig RW, Bingisser R, Nickel CH (2017). Performance of the modified Richmond Agitation Sedation Scale in identifying delirium in older ED patients. Am J Emerg Med.

