# Supplementary material for: Delirium detection in older acute medical inpatients: a multicentre prospective comparative diagnostic test accuracy study of the 4AT and the confusion assessment method
Source: BMC Med. 2019 Jul 24;17:138. doi: 10.1186/s12916-019-1367-9 (PMC6651960; doi:10.1186/s12916-019-1367-9)
Supplement: Supplementary file 1 — Table S1. Baseline characteristics by index test (4AT or CAM). Legend: numbers are n (%) or mean (SD). (DOCX 13 kb) [file 12916_2019_1367_MOESM1_ESM.docx]

**Additional Table 1: Baseline Characteristics by Index Test (4AT or CAM)**

|  | *4AT (N=395)* | *CAM (N=390)* |
| --- | --- | --- |
| **Age (Years)** | | |
| Mean (SD) | 81.0 (6.0) | 81.8 (6.8) |
| Median [Q1-Q3] | 81.0 [77.0-85.0] | 82.0 [76.0-87.0] |
| **Gender** | | |
| Female | 214 (54.2%) | 222 (56.9%) |
| Male | 181 (45.8%) | 168 (43.1%) |
| **Dementia Diagnosis** | | |
| Missing | 0 (.) | 1 (.) |
| No | 368 (93.2%) | 345 (88.7%) |
| Yes | 27 (6.8%) | 44 (11.3%) |
| **Location of First Assessment** | | |
| Acute Unit | 334 (84.6%) | 331 (84.9%) |
| Emergency Department | 25 (6.3%) | 28 (7.2%) |
| Hospital Ward | 36 (9.1%) | 31 (7.9%) |
| **Delirium** | | |
| No | 346 (87.6%) | 344 (88.2%) |
| Yes | 49 (12.4%) | 46 (11.8%) |
| Numbers are n (%) or mean (SD). | | |
