# Supplementary material for: Delirium detection in older acute medical inpatients: a multicentre prospective comparative diagnostic test accuracy study of the 4AT and the confusion assessment method
Source: BMC Med. 2019 Jul 24;17:138. doi: 10.1186/s12916-019-1367-9 (PMC6651960; doi:10.1186/s12916-019-1367-9)
Supplement: Supplementary file 2 — Table S2. Performance of various cut points of 4AT for diagnosis of delirium. Legend: numbers are estimates (95% CI). Abbreviations: CI, confidence interval; PPV, positive predictive value; NPV, negative predictive value. Youden’s Index is equal to sensitivity + specificity − 1, a value of zero indicates no value, and a value of 1 indicates a perfect test. (RTF 77 kb) [file 12916_2019_1367_MOESM2_ESM.rtf]

Additional Table 2: Performance of various cut points of 4AT for diagnosis of delirium


Cutpoint on 4AT, >=	Sensitivity	Specificity	PPV	NPV	Youden's Index	
3	79.59% (65.66%,89.76%)	92.42% (89.09%,94.99%)	60.00% (47.10%,71.96%)	96.94% (94.45%,98.52%)	0.72	
4	75.51% (61.13%,86.66%)	94.46% (91.48%,96.63%)	66.07% (52.19%,78.19%)	96.43% (93.84%,98.14%)	0.70	
2	83.67% (70.34%,92.68%)	85.71% (81.56%,89.24%)	45.56% (35.02%,56.40%)	97.35% (94.85%,98.85%)	0.69	
5	65.31% (50.36%,78.33%)	96.50% (93.97%,98.18%)	72.73% (57.21%,85.04%)	95.11% (92.29%,97.13%)	0.62	
1	87.76% (75.23%,95.37%)	69.68% (64.51%,74.50%)	29.25% (22.05%,37.31%)	97.55% (94.75%,99.10%)	0.57	
6	55.10% (40.23%,69.33%)	98.54% (96.63%,99.53%)	84.38% (67.21%,94.72%)	93.89% (90.89%,96.13%)	0.54	
7	46.94% (32.53%,61.73%)	99.42% (97.91%,99.93%)	92.00% (73.97%,99.02%)	92.92% (89.79%,95.32%)	0.46	
8	36.73% (23.42%,51.71%)	99.42% (97.91%,99.93%)	90.00% (68.30%,98.77%)	91.67% (88.38%,94.27%)	0.36	
9	30.61% (18.25%,45.42%)	100.00% (98.93%,100.00%)	100.00% (78.20%,100.00%)	90.98% (87.63%,93.67%)	0.31	
10	26.53% (14.95%,41.08%)	100.00% (98.93%,100.00%)	100.00% (75.29%,100.00%)	90.50% (87.09%,93.26%)	0.27	
11	22.45% (11.77%,36.62%)	100.00% (98.93%,100.00%)	100.00% (71.51%,100.00%)	90.03% (86.57%,92.84%)	0.22	
12	20.41% (10.24%,34.34%)	100.00% (98.93%,100.00%)	100.00% (69.15%,100.00%)	89.79% (86.31%,92.64%)	0.20	
Numbers are estimates (95% CI). Youden's Index is equal to sensitivity+specificity-1, a value of zero indicates no value, and a value of 1 indicates a perfect test.
Abbreviations: CI, confidence interval; PPV, positive predictive value; NPV, negative predictive value.
			Numbers are estimate (95% CI).
 Abbreviations: CI, confidence interval; PPV, positive predictive value; NPV, negative predictive value;
OR, odds ratio; N/A, not applicable.
Odds Ratio not applicable due to zero events in one group.
 Youden's Index is equal to sensitivity+specificity-1, a value of zero indicates no value, and a value of 1
indicates a perfect test.	
 
