# Supplementary material for: Delirium detection in older acute medical inpatients: a multicentre prospective comparative diagnostic test accuracy study of the 4AT and the confusion assessment method
Source: BMC Med. 2019 Jul 24;17:138. doi: 10.1186/s12916-019-1367-9 (PMC6651960; doi:10.1186/s12916-019-1367-9)
Supplement: Supplementary file 3 — Table S3. Sensitivity analysis of diagnostic test accuracy of 4AT versus CAM for diagnosis of delirium assuming all indeterminates are delirium present. Legend: numbers are estimate (95% CI). Difference in proportions is for 4AT-CAM. Abbreviations: CI, confidence interval; PPV, positive predictive value; NPV, negative predictive value; OR, odds ratio. Youden’s Index is equal to sensitivity + specificity − 1, a value of zero indicates no value, and a value of 1 indicates a perfect test. (DOCX 14 kb) [file 12916_2019_1367_MOESM3_ESM.docx]

**Additional Table 3: Sensitivity analysis of diagnostic test accuracy of 4AT versus CAM for diagnosis of delirium assuming all indeterminates are delirium present**

|  | **Sensitivity** | | **Specificity** | **PPV** | **NPV** | **Youden's Index** |  |  |
| --- | --- | --- | --- | --- | --- | --- | --- | --- |
| **4AT (>3), *% (95% CI)*** | | 64.2% (51.5 to 75.5%) | 94.5% (91.5 to 96.6%) | 69.4% (56.4 to 80.4%) | 93.1% (89.9 to 95.5%) | 0.59 |  |  |
| **CAM Positive, *% (95% CI)*** | | 32.7% (20.7 to 46.7%) | 99.7% (98.4 to 100.0%) | 94.7% (74.0 to 99.9%) | 90.2% (86.8 to 93.0%) | 0.32 |  |  |
|  | |  |  |  |  |  |  |  |
| **Difference in Proportions** | | 31.5% (13.8 to 47.6%) | -5.25% (-12.7 to 2.23%) | -25.4% (-49.4 to 0.30%) | 2.89% (-4.39 to 10.2%) |  |  |  |
| ***P* value** | | 0.0010 | <0.0001 | 0.0318 | 0.1813 |  |  |  |

Numbers are estimates (95% CI). Youden's Index is equal to sensitivity+specificity-1, a value of zero indicates no value, and a value of 1 indicates a perfect test. The Difference in Proportions is 4AT-CAM for for each of the tabulated measures of diagnostic accuracy, accompanied by the corresponding P-value from the Fisher’s exact test comparing proportions. Abbreviations: CI, confidence interval; PPV, positive predictive value; NPV, negative predictive value.
