# Supplementary material for: Delirium detection in older acute medical inpatients: a multicentre prospective comparative diagnostic test accuracy study of the 4AT and the confusion assessment method
Source: BMC Med. 2019 Jul 24;17:138. doi: 10.1186/s12916-019-1367-9 (PMC6651960; doi:10.1186/s12916-019-1367-9)
Supplement: Supplementary file 4 — Table S4. Sensitivity analysis of diagnostic test accuracy of 4AT versus CAM for diagnosis of delirium assuming all indeterminates are delirium absent. Legend: numbers are estimate (95% CI). Difference in proportions is for 4AT-CAM. Abbreviations: CI, confidence interval; PPV, positive predictive value; NPV, negative predictive value; OR, odds ratio. Youden’s Index is equal to sensitivity + specificity − 1, a value of zero indicates no value, and a value of 1 indicates a perfect test. (DOCX 14 kb) [file 12916_2019_1367_MOESM4_ESM.docx]

| **Additional Table 4: Sensitivity analysis of diagnostic test accuracy of 4AT versus CAM for diagnosis of delirium assuming all indeterminates are delirium absent**   \|  \| \| **Sensitivity** \| \| **Specificity** \| \| **PPV** \| \| **NPV** \| \| **Youden's Index** \| \| --- \| --- \| --- \| --- \| --- \| --- \| --- \| --- \| --- \| --- \| --- \| \| **4AT (>3), *% (95% CI)*** \| \| 75.5% (61.1 to 86.7%) \| \| 93.1% (90.0 to 95.5%) \| \| 59.7% (46.5 to 72.0%) \| \| 96.6% (94.1 to 98.2%) \| \| 0.69 \| \| **CAM Positive, *% (95% CI)*** \| \| 40.5% (25.6 to 56.7%) \| \| 99.4% (98.0 to 99.9%) \| \| 89.5% (66.9 to 98.7%) \| \| 93.4% (90.4 to 95.7%) \| \| 0.40 \| \|  \| \|  \| \|  \| \|  \| \|  \| \|  \| \| **Difference in Proportions** \| \| 35.0% (14.7 to 53.2%) \| \| -6.36% (-13.7 to 1.04%) \| \| -29.8% (-53.6 to -4.37%) \| \| 3.17% (-4.11 to 10.4%) \| \|  \| \| ***P* value** \| \| 0.0012 \| \| <.0001 \| \| 0.0242 \| \| 0.0629 \| \|  \| \|  \|  \| \|  \| \|  \|  \|  \| \|   Numbers are estimates (95% CI). Youden's Index is equal to sensitivity+specificity-1, a value of zero indicates no value, and a value of 1 indicates a perfect test. The Difference in Proportions is 4AT-CAM for for each of the tabulated measures of diagnostic accuracy, accompanied by the corresponding P-value from the Fisher’s exact test comparing proportions. Abbreviations: CI, confidence interval; PPV, positive predictive value; NPV, negative predictive value. |
| --- | --- | --- | --- | --- | --- | --- | --- | --- | --- | --- | --- | --- | --- | --- | --- | --- | --- | --- | --- | --- | --- | --- | --- | --- | --- | --- | --- | --- | --- | --- | --- | --- | --- | --- | --- | --- | --- | --- | --- | --- | --- | --- | --- | --- | --- | --- | --- | --- | --- | --- | --- | --- | --- | --- | --- | --- | --- | --- | --- | --- | --- | --- | --- | --- | --- | --- | --- | --- | --- | --- | --- | --- | --- | --- | --- |
