# Supplementary material for: Delirium detection in older acute medical inpatients: a multicentre prospective comparative diagnostic test accuracy study of the 4AT and the confusion assessment method
Source: BMC Med. 2019 Jul 24;17:138. doi: 10.1186/s12916-019-1367-9 (PMC6651960; doi:10.1186/s12916-019-1367-9)
Supplement: Supplementary file 5 — Table S5. Diagnostic test accuracy of 4AT versus CAM for diagnosis of delirium, assuming test scored delirium present for those with a missing 4AT or CAM score. Legend: numbers are estimate (95% CI). Difference in proportions is for 4AT-CAM. Abbreviations: CI, confidence interval; PPV, positive predictive value; NPV, negative predictive value; OR, odds ratio. Youden’s Index is equal to sensitivity + specificity − 1, a value of zero indicates no value, and a value of 1 indicates a perfect test. (DOCX 14 kb) [file 12916_2019_1367_MOESM5_ESM.docx]

| **Additional Table 5: Diagnostic test accuracy of 4AT versus CAM for diagnosis of delirium, assuming test scored delirium present for those with a missing 4AT or CAM score**   \|  \| \| **Sensitivity** \| **Specificity** \| **PPV** \| **NPV** \| **Youden's**  **Index** \| \| --- \| --- \| --- \| --- \| --- \| --- \| --- \| \| **4AT (>3), *% (95% CI)*** \| \| 75.5% (61.1 to 86.7%) \| 93.6% (90.5 to 96.0%) \| 62.7% (49.2 to 75.0%) \| 96.4% (93.8 to 98.1%) \| 0.69 \| \| **CAM Positive, *% (95% CI)*** \| \| 45.7% (30.9 to 61.0%) \| 99.1% (97.5 to 99.8%) \| 87.5% (67.6 to 97.3%) \| 93.2% (90.1 to 95.5%) \| 0.45 \| \|  \| \|  \|  \|  \|  \|  \| \| **Difference in Proportions** \| \| 29.9% (9.51 to 47.9%) \| -5.49% (-13.0 to 2.09%) \| -24.8% (-47.0 to -1.04%) \| 3.26% (-4.14 to 10.6%) \|  \| \| ***P* value** \| \| 0.0034 \| 0.0001 \| 0.0342 \| 0.0629 \|  \| \|  \|  \| \|  \|  \|  \|  \| |
| --- | --- | --- | --- | --- | --- | --- | --- | --- | --- | --- | --- | --- | --- | --- | --- | --- | --- | --- | --- | --- | --- | --- | --- | --- | --- | --- | --- | --- | --- | --- | --- | --- | --- | --- | --- | --- | --- | --- | --- | --- | --- | --- | --- | --- | --- | --- | --- | --- | --- |

Numbers are estimates (95% CI). Youden's Index is equal to sensitivity+specificity-1, a value of zero indicates no value, and a value of 1 indicates a perfect test. The Difference in Proportions is 4AT-CAM for for each of the tabulated measures of diagnostic accuracy, accompanied by the corresponding P-value from the Fisher’s exact test comparing proportions. Abbreviations: CI, confidence interval; PPV, positive predictive value; NPV, negative predictive value.
